# MicroRNA miR-4709-3p targets Large Tumor Suppressor Kinase 2 (LATS2) and induces obstructive renal fibrosis through Hippo signaling

**DOI:** 10.1080/21655979.2021.2002493

**Published:** 2021-12-21

**Authors:** Zexiang Jiang, Weiping Xia, Guoyu Dai, Bo Zhang, Yang Li, Xiang Chen

**Affiliations:** Department of Urology Surgery, Xiangya Hospital Central South University, Changsha City, China

**Keywords:** Chronic kidney disease (CKD), Hippo signaling, miR-4709-3p, unilateral ureteric obstruction (UUO)

## Abstract

Obstructive renal fibrosis is the consequence of abnormal extracellular matrix assembly, which eventually results in renal failure, acute, and end‑stage renal infection. MicroRNAs (miRNAs), a particular category of small RNAs, modulate the expression of genes post-transcriptionally and regulate biological activities, including fibrogenesis. The study probed to estimate the key functions of miR-4709-3p in obstructive renal fibrosis. This investigation used TGF-β1 stimulated HK-2 in-vitro model, unilateral ureteral occlusion (UUO) mice model, and human Diabetic nephropathy (DN) and Renal interstitial fibrosis (RIF) specimens to depict the abundance of the miR-4709-3p level using FISH and RT-qPCR. MiR-4709-3p mimics and inhibitors were utilized to evaluate the functions of miR-4709-3p in-vitro. Luciferase assay was exploited to verify miR-4709-3p and LATS2 3ʹUTR binding. Finally, to depict the functions of miR-4709-3p in-vivo, the UUO model was injected with miR-4709-3p inhibitors. Results exhibited the upregulation of miR-4709-3p in UUO-induced in-vivo model, TGF-β1 stimulated HK-2, and human RIF and DN samples. Moreover, it was determined that modulating miR-4709-3p regulated the level of fibrosis markers. Luciferase assay miR-4709-3p modulates renal fibrosis by targeting LATS2. Finally, it was found that miR-4709-3p regulates obstructive renal fibrosis through the Hippo signaling pathway. Overall, the study concludes that aberrant miR-4709-3p expression plays an essential function in the renal fibrosis progression, and miR-4709-3p overexpression could advance obstructive renal fibrosis via LATS2 targeting in Hippo signaling pathway. Therefore, miR-4709-3p inhibition may be a potential renal fibrosis therapy target.

## 1. Introduction

Renal fibrosis is the fibrotic matrix deposition and scar formation as a response to persistent or severe injury^1^. Even though renal fibrosis is involved in wound healing, persistent fibrosis may destroy tissue structure and organ performance, finally resulting in renal failure^2^. Kidney Chronic injury promotes various pathological changes, such as epithelial-mesenchymal transition (EMT), endothelial–mesenchymal transition (EndoMT), and fibroblasts and pericytes activation^3^. The characteristics of EMT are the loss of intracellular adhesion, for instance, E-cadherin, and the mesenchymal markers’ acquisition, such as αSMA, fibronectin, fibroblast-specific protein 1 (FSP1), vimentin, and collagen^4^. EndoMT is a special subset of EMT occurring in endothelial cells, with similarity to EMT^5^. Pericytes and fibroblasts are often described as the major myofibroblasts’ origin, whose activation mainly induces the formation of myofibroblasts^3^. These pathological occurrences lead to renal cells transformation into myofibroblasts, exerting their profibrotic function by producing collagen I, III, and IV, laminin, and fibronectin resulting to the accumulation of extracellular matrix (ECM) and eventually leading to tubulointerstitial fibrosis

The disease covers four stages: fibrogenic level, activation, expansion stage, and progression^6^. In the last few years, potential drugs have been identified, such as SGLT2 inhibitor in protecting the kidneys^7^, while endothelin antagonists have also been reported to retard fibrosis^8^. Further, some results in mouse models include DPP-4 inhibitor linagliptin, empagliflozin, SIRT3, JAK-stat inhibitors, glycolysis inhibitors, ACE inhibitors, ARBs, and peptide AcSDKP; and they have all confirmed a protective role in renal fibrosis^9^. An essential renal fibrosis mediator is the signaling of transforming growth factor-β (TGF-β), which occurs through the extracellular matrix induction, leading to scarring in the renal system^10^. Mitogen‑activated protein kinase (MAPK) is essential in renal fibrosis’s extracellular matrix since it is downstream of TGF-β. The involved molecular mechanisms, such as the signaling of MAPK and TGFβ, have been proposed as the possible renal fibrosis treatment targets^11^.

MicroRNAs (miRNAs) are short non-coding endogenous RNAs of approximately 17–21 nucleotides, important for gene expression regulation by modifying post‑translation and initiation of mRNA degradation, affecting various cellular and molecular processes^12^. MiRNAs play various roles in different types of cancers^13^ and metabolic diseases^14^. MiRNAs that play a role in regulating fibro-proliferative diseases have been referred to as fibromiR, and miRNAs roles in the kidney’s physiological functioning have been described; for instance, miR-150 has been associated with induced renal tissue fibrosis in lupus nephritis via SOCS1 targeting^15,16^. MiR-181 plays a suppressor role in renal fibrosis by weakening profibrotic marker expression^12^. Several other miRNAs, such as miR-21, miR-29, and miR-192, have been reported as crucial transforming growth factors (TGF-β) signaling mediators in the renal cells^17,18^. Additionally, treatment with Lisinopril, angiotensin-converting-enzyme inhibitor, caused the kidney’s antifibrotic effect via inhibiting miR-324-3p-dependent suppression of prolyl endopeptidase (POP), a serine peptidase involved in the synthesis of the endogenous antifibrotic peptide AcSDKP, essential in ECM homeostasis^19^. Secretion of MiR-4709-3p is elevated in chronic kidney disease (CKD) and unilateral ureteric obstruction (UUO) in human samples^20^. Consequently, normalization of these miRNAs’ expressions is important in alleviating fibrosis; hence, these proteins are potential therapeutic targets for renal fibrosis^18^. MiRNAs may play a profibrotic or antifibrotic role based on the cell type of the kidney. The altered miRNAs level in EMT and EndMT processes regulates the synthesis and accumulation of fibroblast in the kidney. MiR-29 family clusters play a major antifibrotic role in kidney fibrosis associated with *Smad*-dependent and *Smad*-independent pathways; hence, they have renal protective action in EndMT and associated renal fibrosis^21^. Similarly, miR-let-7 family clusters also have an antifibrotic role in renal fibrosis, and AcSDKP suppresses EndMT-driven renal fibrosis via ameliorating miR-let-7 family clusters^19^. Finally, drugs DPP-4 inhibitor and peptide AcSDKP show renal protection by regulating crosstalk regulation between miR-29 and mR-let-7^19^.

Similar to TGF-β, the hippo signaling pathway transfers plasma membrane signals to the nucleus, and the consequence is an altered expression of genes that regulate the cells’ survival^22^. The Hippo pathway exerts significant effects on the organ size regulation, embryonic development, tumorigenesis, epithelial to mesenchymal transition (EMT), and homeostasis of the stem cell^23^. The Hippo signaling exerts vital, complex, and vigorous core function via the LATS1 or LATS2 (Lats1/2), which functions as kinases and regulators of MOB1, YAP1, and MST1/2^24^. The LATS2 are in the LATS/NDR kinase family, and they are essential for encoding serine/threonine-protein kinase, which belongs to AGC, a well-known protein kinase^25^. The hippo signaling pathway is also important in kidney infections’ pathophysiological process, especially during the repair. Its role is confirmed by the increased activation of YAP following kidney injury^26^.

As a tumor inhibitor, LATS2 is crucial for duplicating the centrosome and mitotic fidelity maintenance since it has a protein that localizes into the centrosomes during interphase and in the late metaphases^27^. LATS2 may downregulate cells’ proliferation at the G1/S transition by suppressing cyclin E/CDK2 kinase^28^ and initiating apoptosis through the suppression of apoptosis inhibitors, such as Bcl-xL and Bcl-2^29^. After the Hippo activation, LATS1/2 are phosphorylated by MST1/2. Subsequently, the stimulated Lats1/2, together with the Mob1 tumor inhibitor, phosphorylates, and deactivates TAZ and YAP transcriptional coactivators through degradation mediated by proteasome and their cytoplasmic retention^30^. YAP and TAZ can, however, be recruited directly to their target promoters through the TEAD/TEF transcription modulators binding as an alternative of binding directly to DNA^29^ in which they regulate the transcription of proteins necessary for cell proliferation, EMT, apoptosis, survival, expansion of cancer stem cell and differentiation^31^. The Hippo Pathway activity, particularly TAZ/YAP, can be controlled by growth factors, cell–cell junction generated signals, and tissue architecture^32^. It has also been shown that Hippo pathway dysregulation is associated with EMT and the development of cancer, mainly induced by TAZ and YAP^33^. Even though numerous miRNAs, like miR-93, miR-181b, and miR-372, are targeting LATS2 and are directly involved in Hippo signaling Pathway in several cancer types and kidney infections^33^, the role of miRNAs in kidney fibrosis is less clear, and the potential mechanisms of miR-4709-3p/LATS-2 regulating renal fibrosis remain unknown. The present investigation hypothesized that miR-4709-3p suppresses obstructive renal fibrosis via Hippo signaling by targeting Large Tumor Suppressor Kinase 2 (LATS2). The research aimed to determine the expression of MiR-4709-3p in the obstructive renal fibrosis model, determine the effects of miR-4709-3p on renal fibrosis markers, assess the protein target of miR-4709-3p in renal fibrosis, and establish the association between miR-4709-3p and TGF-β1-induced renal fibrosis. Finally, the link between MiR-4709-3p and the Hippo signaling pathway, and the effects of In-vivo inhibition of miR-4709-3p on UUO-induced obstructive renal fibrosis was established.

## 2. Materials and Methods

### 2.1. Human specimens

For our study, we collected 14 healthy kidney specimens and 19 Diabetic nephropathy (DN) specimens, and 14 renal interstitial fibrosis (RIF). All the procedures were permitted by our institute and based on the Declaration of Helsinki. The authorization number for the human study was AU100AC. The characteristics are described in Table 1.

**Table 1.** Characteristics of human specimens used in this study characteristics of human specimens used in this study

Clinical features	DN (n = 19)	RIF (n = 14)	Healthy (n = 14)
Age (years in mean)	65.09	67.04	68.76
Sex (male/female)	12/7	9/5	8/6
UAER (mean, µg/min)	212.23	249	23.76
SCR (mean, µmol/l)	112.63	140	69.82
BUN (mean, mmol/l)	12.15	15	6.02

UAER, urine albumin excretion rate; BUN, blood urea nitrogen; Scr, serum creatinine; DN, diabetic nephropathy; RIF, renal interstitial fibrosis

### 2.2. HK-2 cell culture and transfection

The HK-2, which is recognized as immortal proximal tubule epithelial cell line, was acquired from the institutional biobank. HK-2 cells had been grown and sustained in widely available sterilized DMEM medium augmented in 10% fetal bovine serum. Cell incubation was done in a humidified chamber with 37°C internal temperature and 5% CO_2_. MiR-4709-3p mimics, silencers, and their respective NC (negative controls) were obtained from RiboBio (Guangzhou, China). Transfection of HK-2 cells was done using miR-4709-3p mimics or inhibitor (100 nM), or their respective NC, with Lipofectamine 2000 reagent (Invitrogen, USA) for 24 hours following the guidelines of the manufacturer. HK-2 cells were transfected using varying doses (0, 0.5, 2.5, and 5) of TGF-β1 (ng/ml) to induce renal injury in HK-2 cells and cultured in the same manner^34^.

### 2.3. Lentivirus for LATS2 overexpression

LATS2 sequence synthesis amplified and implanted into a pLVX-IRES-PURO plasmid, a lentiviral system for overexpression of proteins (GenePharma). Later, HK-2 cells were introduced with lenti-LATS2 (LATS2-OE) and other commonly available pLP/VSVG, pLP1, pLP2 packaging mixture of plasmids for 72 hours. The viruses were then garnered and supplemented into the kidney HK-2 cells together with 6 μg/ml polybrene. After the transduction of 72 hours, puromycin (2.5 μg/ml) was used to select the stable LATS 2 overexpressed cells.

### 2.4. Assessment of RNA integrity and purity

Absorbance measurement was done using a nanodrop 1000 spectrometer (Thermo Fisher Scientific, Canada) at 260 nm (A260) to determine RNA concentration. The purity of RNA was established through the ratio of A260 to A280, and only the samples with a ratio exceeding 1.8 were preserved for further analyses. The integrity of RNA was determined through an Agilent 2100 Bioanalyzer through Agilent RNA 6000 Nano Kit (Agilent Technologies, Ontario, Canada). The presence of any inhibitor was assessed using the Solaris RNA Spike Control kit (Thermo Fisher Scientific, # K-002200-C1-100) in RNA samples subsets. An approach based on PCR was used to assess gDNA contamination in RNA samples^35^. All the samples assessed were free from inhibitors and gDNA contamination.

### 2.5. RT-qPCR analysis

The HK-2 cells transfection was done using miR-4709-3p mimics or inhibitors for 6 hours and later exposed to TGF-β1 (5 ng/ml) for 48 hours. The total RNA was then extracted from HK-2 cells using TRIzol reagent (Invitrogen/Thermo Fisher Scientific) following the manufacture’s guideline. cDNAs processing was done by the reverse transcription of RNA using the GoScriptTM Reverse Transcription kit (Promega). Later, qPCR was analyzed using the SYBR® Premix Ex Taq™ II (Takara, Japan). GAPDH and U6 were utilized as the analysis normalization for the abundances of miR-4709-3p and other mRNAs, respectively, as used elsewhere^36^. The primers’ sequences are described in Table 2. All experiments were carried out in triplicates, and mRNA expressions were determined by the 2^−ΔΔCt^ method.

**Table 2.** List of primers used in this study

List of Primers used in this study		
Gene	Forward Primer	Reverse Primer
Hsa-α-SMA	AAGAGCATCCCACCCTGC	TAGCCACATACATGGCTGGG
Hsa-FN	ACAACACCGAGGTGACTGAG	GGACACAACGATGCTTCCTGA
Hsa-Col1	GTGGATACGCGGACTTTG	TCCATCATACTGAGCAGCA
Mmu-α-SMA	CCAACCGGGAGAAAATGA	CAGACGCATGATGGCAT
Mmu-FN	GTCTCCTGGGAGAGGAGC	TGATCAGCATGGACCACT
Mmu-Col1	GGTCCTGATGGCAAAAC	TCCATCTTTGCCAGCAGGA
LATS2	AGGCCAAAGACTTTTCCTGC	CACGTACACAGGCTGGCAGC
Mmu-β-Actin	CAGCTGAGAGGGAAATCGTG	CGTTGCCAATAGTGATGACC
Hsa-β-Actin	ACCATTGGCAATGAGCGGTTC	GGTCTTTGCGGATGTCCACGT
miR-4709-3p	Qiagen (Cat#MS00039914)	
snRNA RNU6B	Qiagen (Cat#MS00033740)	

### 2.6. Western blot

Western blot assay was performed as previously described^37^. Briefly, total proteins quantification was done through the BCA method (Beyotime, Shanghai, China). Subsequently, proteins (30 μg) were separated using 10% sodium dodecyl sulfate-polyacrylamide gel electrophoresis, followed by a transfer into a membrane of polyvinylidene difluoride (PVDF) (Thermo Fisher Scientific). The membrane was later blocked for 1 hour using 5% skimmed milk in TBST, incubated using primary antibodies against Collagen I, α-SMA, Fibronectin, P-Taz, Taz, P-Yap, Yap, LATS2, and β-actin at 4°C overnight. Subsequently, the membrane was incubated for 1 hr at room temperature using secondary antibodies (1:5000, Abcam). Finally, the band detection was done using the ECL Chemiluminescent Substrate Kit (Thermo Fisher Scientific). The GAPDH was utilized as an internal control.

### 2.7. Bioinformatics tools

TargetScan (http://www.targetscan. org) and PicTar (http://pictar.mdc-berlin.de) were utilized to analyze potential miR-4709-3p binding sites on the target genes. The consistency of analysis and predictions through these websites confirms their reliability.

### 2.8. Dual-luciferase reporter assay

The nucleotide sequences of LATS2 along with the predicted binding sites of miR-4709-3p were sub-cloned into the vector (pmirGLO) having wild-type (WT)-LATS2 or mutant (MT)-LATS2. HK-2 cells were then co-transfected using plasmids (WT-LATS2 or MT-LATS2) and miR-4709-3p mimics with Lipofectamine 2000. Luciferase activity in the cell lysates was later determined through a Dual-Luciferase Reporter Assay System (Promega, USA) after 48 hours as per the manufacturer’s guidelines^36^.

### 2.9. Animal study

C57BL/6 mice (eight weeks old, 20–25 g in weight) were obtained from Xiangya Hospital of Central South University institutional animal care facility (XHCA1/B21/04 C). All the experiments involving animals were undertaken after Xiangya Hospital of Central South University Institutional Ethical Committee’s approval and under the strict adherence of the National Institutes of Health Guide for laboratory animals’ care and use. The 24 experimental mice were arbitrarily separated equally into 4 groups with 6/group: blank, unilateral ureteral occlusion (UUO), UUO + Negative control (NC), UUO + miR-4709-3p inhibitors groups.

The UUO animals were obtained using a previous procedure^38^. Summarily, experimental mice were anesthetized intraperitoneally using pentobarbital (50 mg/kg). Exposure of the left ureter was done after laparotomy of the abdominal midline. Next, left ureter obstruction was done by 2-point ligations using silk sutures (4–0). Openings were later fastened in coatings. In the Sham group, the mice were also exposed to abdominal midline laparotomy. However, the left ureter was left unobstructed. MiR-4709-3p inhibitor (50 nM) was administered through an injection in the tail vein daily as described previously^39^. After 4 weeks, the animals were sacrificed, and renal tissues were harvested.

### 2.10. *Fluorescence* In Situ *Hybridization*

*In situ* hybridization assay was done using labeled oligo-sequences of miR-4709-3p and its control sequences, as previously described elsewhere^40^. Briefly, paraffin-embedded specimen sections from kidney tissues of the experimental mice were deparaffinized then rehydrated. After a wash in PBS, the sections were incubated for 10 minutes using Proteinase K (20 μg/mL) at 25°C. The samples were then incubated using a fixative of 1-(3-dimethylaminopropyl)-3-ethylcarbodiimide hydrochloride (EDC) for 30 min. Next, slides were hybridized for 2 h at 60°C using an oligonucleotide probe complementary labeled with a 5′-digoxigenin (DIG) (50 nM) to miR-4709-3p or its control probe. Washing of the sections was then done at 60°C in the washing buffer I, containing saline sodium citrate-SSC-(5×), washing buffer II (SSC-1×), and the washing buffer III (SSC-0.2×), respectively. Later, the sections were incubated in a blocking solution for 30 minutes at room temperature and stained for 1.5 h using anti-digoxigenin-peroxidase (POD) (Roche, USA). After washed in Tris and NaCl (TNT) solutions, the sections were incubated in the dark using TSA Plus Fluorescein kit and mounted using antifade mounting medium and DAPI nuclei visualization (Invitrogen, Oregon, USA). The Slides were finally observed on ZEISS confocal microscope. MiR-4709-3p Fluorescence quantification was determined using Image-Pro Plus software.

### 2.11. Immunohistochemistry (IHC)

Immunohistochemistry was performed as previously described^41^. Summarily, kidney samples from mice were fixed in formaldehyde (4% (w/v)), embedded in paraffin, then cut into 3 μm tissue sections. After deparaffinization, PT Link (Dako, Santa Clara, CA) containing 10 mM citrate sodium buffer was used to expose the sections. Samples treatment with 3% H2O2 was done to block endogenous peroxidase. Tissue sections of the kidney were incubated in bovine serum albumin (BSA) (4%), and serum (8%) in a 1x wash buffer. The samples were later incubated at 4°C using the primary antibodies, LATS2, α-SMA, Col1, Fibronectin, Taz, p-TAz, Yap, and p-Yap (1:1000) overnight. A biotinylated mouse anti-rabbit secondary antibody was used for rabbit primary antibodies detection. The complex analysis was then done using R.T.U Vectastain Elite ABC Kit (Vector Laboratories, CA, USA). Tissue sections of the kidney were stained with 20 μl/ml of 3, 3ʹ-diaminobenzidine (DAB) (Dako), and counterstained using Carazzi’s hematoxylin. Finally, mowiol (Merck Millipore) mounting media was used for mounting the slides, and images were captured by Axioskop2 plus microscope (Zeiss, Germany) at a 20x magnification with the DMC6200 imaging system (Leica Microsystems, Germany).

### 2.12. Statistical analysis

All the experimental data were carried out in triplicates. Data were presented as mean ± SD. Graph-Pad Prism software was used for the analysis of statistics. Tukey’s tests and One-way analysis of variance (ANOVA) were done for multiple groups’ comparison. And student’s t-test for comparison of two groups. The differences were considered significant at *p < 0.05.

## 3. Results

### 3.1. MiR-4709-3p expression is increased in the obstructive renal fibrosis model

Renal fibrosis is the fibrotic matrix deposition and scar formation as a response to persistent or severe injury. An association between miRNAs and renal infections has been established^42^. MiR-4709-3p has been reportedly increased in serum of kidney disease patients^43^. This investigation explored the mechanisms and use of miR-4709-3p on renal fibrosis therapy. The study hypothesized that miR-4709-3p suppresses obstructive renal fibrosis via Hippo signaling by targeting Large Tumor Suppressor Kinase 2 (LATS2).

**Figure 1.**
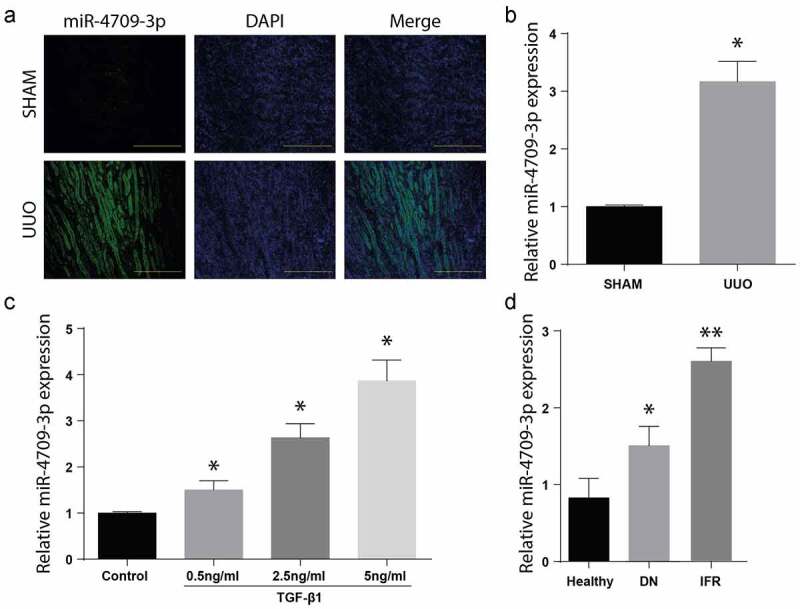
Level of miR-4709-3p upregulated in kidney from human DN specimens, and in-vivo and in-vitro model of obstructive renal fibrosis

To determine the miR-4709-3p role in obstructive renal fibrosis, the mice kidney specimens, with or without renal fibrosis, were assessed through *In Situ* Hybridization assay. A significantly increased miR-4709-3p expression in the mice’s kidney samples with renal fibrosis (UUO), as demonstrated in Figure 1(a). Further, miR-4709-3p mRNA expression in the samples was evaluated through the RT-qPCR. According to the observation, there was a significant upregulation of the miR-4709-3p mRNA level in the UUO model compared to the control group, as shown in Figure 1(b). The miR-4709-3p expression was significantly elevated in a dose-dependent manner, following various doses of TGF-β1 (Figure C). Similarly, miR-4709-3p expression was elevated in the patients’ samples than in the healthy human samples. The observations confirm the increased expression of miR-4709-3p in specimen from patients with renal interstitial fibrosis and diabetic nephropathy (Figure D).

(a) FISH assay showing the expression of miR-4709-3p in kidney samples from UUO-induced mice model.(b) RT-qPCR assay showing miR-4709-3p expression in kidney samples from UUO-induced mice model.(c) RT-qPCR assay showing miR-4709-3p expression in HK-2 cells induced with different doses of TGF-β1.(d) RT-qPCR assay showing miR-4709-3p expression in kidney samples isolated from human DN.(n = 6 each group for UUO-induced mice; n = 14 for healthy individuals; n = 19 for Diabetic nephropathy (DN); data presented as mean ± SD; *p < 0.05; scale bar = 100 µm)

### 3.2. Modulation of miR-4709-3p alters markers of renal fibrosis

**Figure 2.**
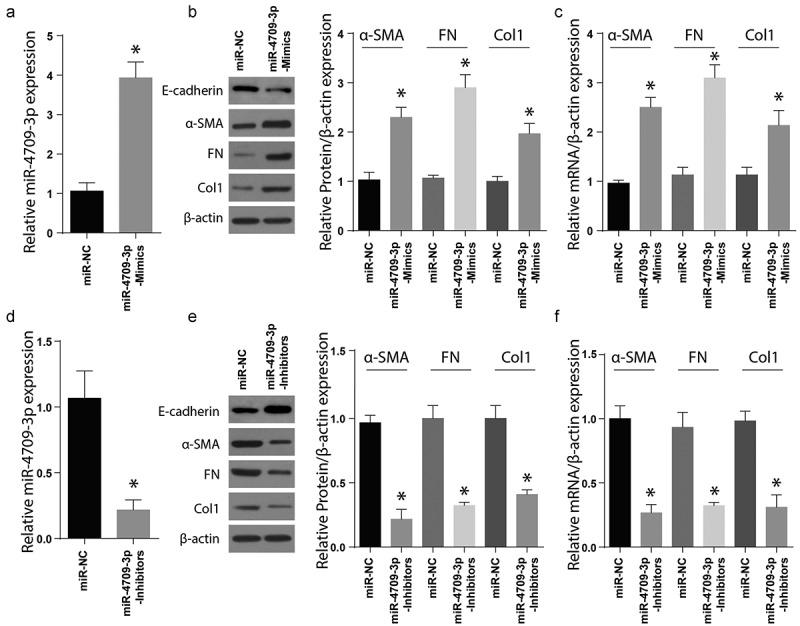
Modulating miR-4709-3p expression regulates renal fibrosis markers

Transforming growth factor-B1 (TGF-B1) regulates the myofibroblastic phenotype, especially in a stiff microenvironment, such as fibrosing tissue^44^. In renal fibrosis, the TGF-B1 has a direct regulatory role in the expression of Col1 and α-SMA^45^. To understand the effect of miR-4709-3p regulation on renal fibrosis, the HK-2 cell line was individually transfected with miR-4709-3p-mimics or the miR-NC. Results exhibited a significant increase in miR-4709-3p level following the transfection of miR-4709-3p mimics compared to miR-NC (Figure 2(a)). Later, the expression of renal fibrosis protein markers, Col1, Fibronectin, α-SMA, and E-cadherin was determined using the Western blot assay. According to the results, Col1, Fibronectin, and α-SMA protein levels were more significantly advanced in the miR-4709-3p mimics-transfected cells than in the miR-NC transfections expressed in Figure 2(b). However, the expression of E-cadherin was reduced in the miR-4709-3p mimics-transfected cells than in the miR-NC transfections. The miR-4709-3p mRNA levels were also determined in cells transfected using miR-4709-3p mimic or miR-NC through RT-qPCR. The results further confirmed an elevated miR-4709-3p mRNA post-transfection using miR-4709-3p-mimics (Figure 2(c)). Next, the HK-2 cells were transfected using miR-4709-3p-inhibitors or the miR-NC, and the renal fibrosis proteins markers were assessed by Western blotting. According to the observations, transfection of miR-4709-3p inhibitors considerably reduced the miR-4709-3p levels in HK-2 cells compared to the miR-NC (Figure 2(d)). Moreover, Col1, Fibronectin, and α-SMA proteins expressions were significantly inhibited after transfection with miR-4709-3p-inhibitors compared to the HK-2 cells transfected with miR-NC. However, the expression of E-cadherin was significantly increased in the miR-4709-3p mimics-transfected cells than in the miR-NC transfections. as shown in Figure 2(e). Finally, the mRNA levels following HK-2 transfection with miR-4709-3p-inhibitors indicated a significant drop compared to transfection with miR-NC, as shown in Figure 2(f). These findings demonstrate that modulation of miR-4709-3p is critical for the development of renal fibrosis.

(a) RT-qPCR assay showing miR-4709-3p expression in HK-2 cells transfected with miR-4709-3p mimics.(b) Western blot analysis exhibiting protein level of renal fibrosis markers in HK-2 cells transfected with miR-4709-3p mimics.(c) RT-qPCR assay depicting mRNA level of renal fibrosis markers in HK-2 cells transfected with miR-4709-3p mimics.(d) RT-qPCR assay showing miR-4709-3p expression in HK-2 cells transfected with miR-4709-3p inhibitors.(e) Western blot analysis exhibiting protein level of renal fibrosis markers in HK-2 cells transfected with miR-4709-3p inhibitors.(f) RT-qPCR assay depicting mRNA level of renal fibrosis markers in HK-2 cells transfected with miR-4709-3p inhibitors.(Data presented as mean ± SD; *p < 0.05)

### 3.3. LATS2 protein is a target of miR-4709-3p in renal fibrosis

**Figure 3.**
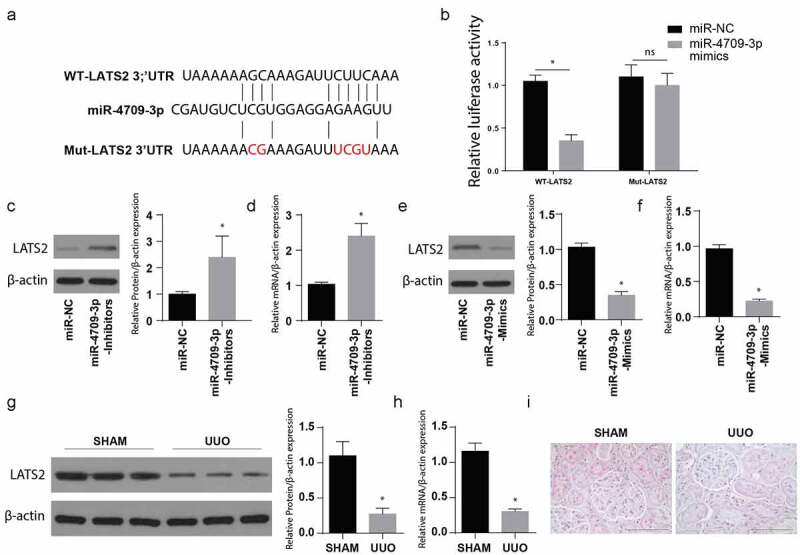
MiR-4709-3p targets LATS2 3ʹUTR and regulates its expression

MiR-4709-3p target gene assessment done through miRNA prediction tool, e.g., TargetScan and PicTar, indicated that LATS2 has a site crucial for binding miR-4709-3p at the 3ʹUTR region, as indicated in Figure 3(a). As a confirmation, a luciferase reporter assay was undertaken to determine the luciferase activity. MiR-4709-3p, its mutant, together with the wild-type LATS2 3ʹUTR were demonstrated in Figure 3(a). The LATS2 WT or Mut-3ʹUTR in HK-2 – luciferase assessment in the HK-2 cells post-transfection using WT LAST2 and the miR-4709-3p-mimics demonstrated a significantly inhibited luciferase activity in cells transfected with WT-LATS2 and miR-4709-3p-mimics (Figure 3(b)). Moreover, co-transfection of Mut-LATS2 and miR-4709-3p mimics showed no significant change in relative luciferase activity.

(a) MiR-4709-3p binding site in the 3ʹUTR of wildtype and mutant LATS2.(b) Relative luciferase activity showing the interaction of LATS2 3ʹUTR with miR-4709-3p.(c) Western blot analysis exhibiting protein level of LATS2 in HK-2 cells transfected with miR-4709-3p inhibitors.(d) RT-qPCR assay depicting mRNA level of LATS2 in HK-2 cells transfected with miR-4709-3p inhibitors.(e) Western blot analysis exhibiting protein level of LATS2 in HK-2 cells transfected with miR-4709-3p mimics.(f) RT-qPCR assay depicting mRNA level of LATS2 in HK-2 cells transfected with miR-4709-3p mimics.(g) Western blot analysis exhibiting protein level of LATS2 in kidney samples from UUO-induced mice model.(h) RT-qPCR assay depicting mRNA level of LATS2 in kidney samples from UUO-induced mice model.(i) IHC assay depicting protein level of LATS2 in kidney samples from UUO-induced mice model.(n = 6 each group; data presented as mean ± SD; *p < 0.05; scale bar = 100 µm)

Later, the Western blotting technique was utilized to evaluate the LATS2 protein expression following the transfection of cells with miR-4709-3p-inhibitors or its miR-NC. The observations indicated a significantly increased LATS2 protein expression following the transfection with miR-4709-3p-inhibitors compared to the miR-NC group, as shown in Figure 3(c). RT-qPCR was also used to determine the LATS2 mRNA expression. The result also indicated an increased LATS2 mRNA expression after the transfection using the miR-4709-3p-inhibitors compared to the miR-NC group, Figure 3(d).

To understand the consequence of miR-4709-3p overexpression, a well-grown HK-2 cell line was incubated with miR-4709-3p-mimics or miR-NC. LATS2 protein and mRNA expressions were then assessed through Western blot and RT-qPCR, respectively. The observations indicated a significantly reduced LATS protein and mRNA following the transfection with miR-4709-3p-mimics, as depicted in Figure 3(e,f), respectively.

Tissues of UUO and the sham (negative control) mice were then obtained, and the LATS2 protein and mRNA expressions were assessed using Western blot and RT-qPCR, respectively. In the observation, LATS2 protein and mRNA were significantly reduced in the UUO animal model compared to the sham group, as shown in Figure 3(g,h). Similarly, the IHC analysis depicted a significantly reduced LATS2 in the UUO model compared to the sham group (Figure 3(i)). Together, the data show that LATS2 expression decreases during obstructive renal fibrosis. Moreover, miR-4709-3p binds to LATS2 3ʹUTR and regulates its expression both at mRNA and the protein level.

### 3.4. MiR-4709-3p accelerates the TGF-β1-induced renal fibrosis in-vitro

**Figure 4.**
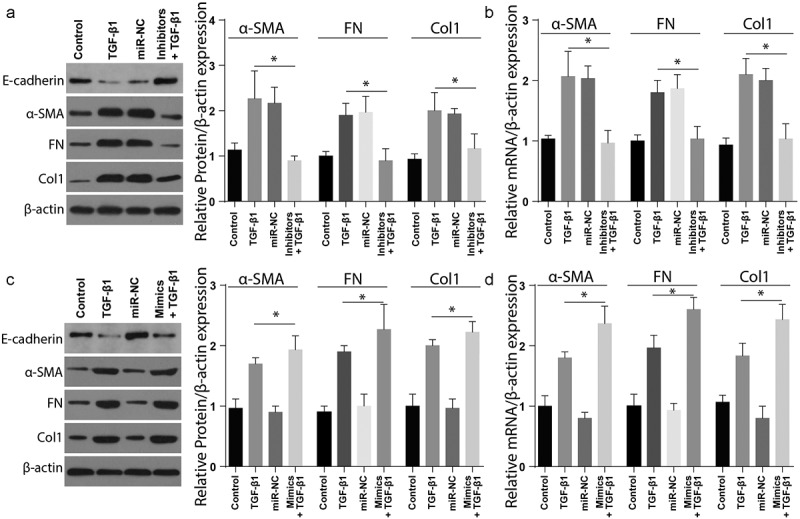
Modulating miR-4709-3p expression regulates TGF-β1 induced renal fibrosis in HK-2 cells

In order to understand the miR-4709-3p role in renal fibrosis in vivo, renal fibrosis markers expression was assessed in the cells co-transfected using TGF-β1, miR-NC, or miR-4709-3p inhibitors and TGF-β1. As demonstrated in Figure 4(a), the inhibition of miR-4709-3p considerably reduced the expression of Col1, Fn, and α-SMA proteins in the LX-2 cells stimulated with TGF-β1. However, miR-4709-3p inhibition significantly increased the expression of E-cadherin in the LX-2 cells stimulated with miR-4709-3p inhibitors and TGF-β1. The mRNA expression analysis through RT-qPCR also demonstrated significant suppression of mRNA following co-transfection with TGF-β1, miR-NC, or miR-4709-3p inhibitors and TGF-β1 TGF-β1, as shown in Figure 4(b).

(a) Western blot analysis exhibiting protein level of renal fibrosis markers in HK-2 cells transfected with TGF-β1 and miR-4709-3p inhibitors.(b) RT-qPCR assay depicting mRNA level of renal fibrosis markers in HK-2 cells transfected with TGF-β1 and miR-4709-3p inhibitors.(c) Western blot analysis exhibiting protein level of renal fibrosis markers in HK-2 cells transfected with TGF-β1 and miR-4709-3p mimics.(d) RT-qPCR assay depicting mRNA level of renal fibrosis markers in HK-2 cells transfected with TGF-β1 and miR-4709-3p mimics.(Data presented as mean ± SD; *p < 0.05)

Additionally, the renal fibrosis markers’ expression was assessed in cells co-transfected with TGF-β and miR-4709-3p-mimics. As depicted in Figure 4(c), Col1, Fn, and α-SMA significantly increased in cells positive for renal fibrosis compared to the sham group. Nevertheless, expression of E-cadherin remarkably reduced in the cells positive for renal fibrosis compared to the sham group. The RT-qPCR examination also showed similar results following transfection with TGF-β and miR-4709-3p-mimics, Figure 4(d). These observations confirm that TGF-β-induced renal fibrosis is modulated by miR-4709-3p in-vitro.

### 3.5. MiR-4709-3p inhibition suppress obstructive renal fibrosis through the Hippo signaling pathway

**Figure 5.**
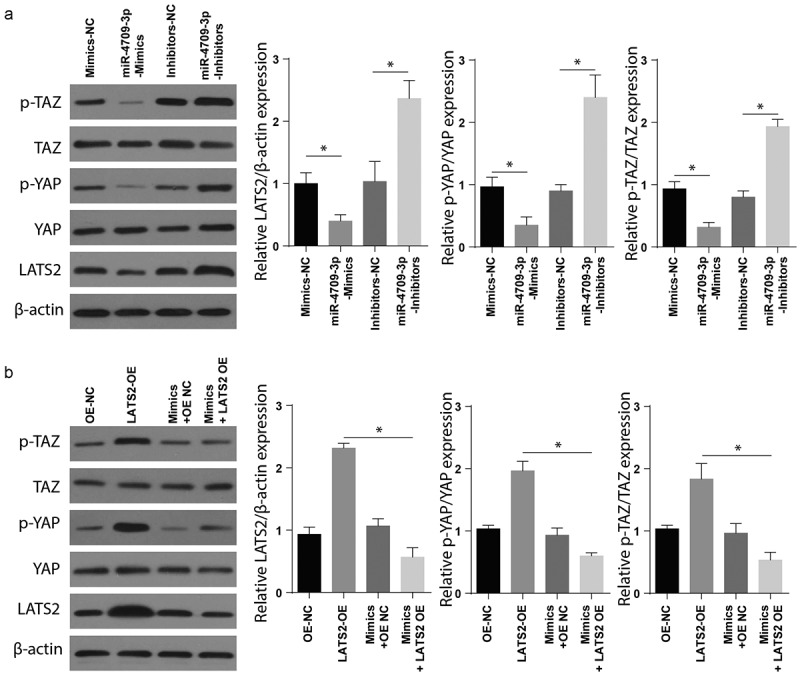
Modulating miR-4709-3p expression regulates Hippo-signaling via LATS2/YAP/TAZ

Finally, to determine the mechanisms involved in modulating obstructive renal fibrosis by miR-4709-3p-mimics, the expression of Hippo signaling markers was assessed following transfection with mimics-NC, miR-mimics, the inhibitors-NC, and miR-4709-3p-inhibitors. The Western blot assay confirmed a significantly elevated p-TAZ, p-YAP, and LATS2 in renal fibrosis samples transfected with miR-4709-3p-inhibitors compared to the sham group (Figure 5(a)). To understand the function of the LATS2 and Hippo signaling pathway, the renal fibrosis positive and sham cells were co-transfected with LATS-OE, OE-NC, Mimics+OE-NC, and mimics+LATS2-OE. The expression of Hippo signaling markers was then studied using Western blot. The results indicated a significantly increased p-TAZ and LATS2 in the renal fibrosis-positive compared to the control (Figure 5(b)). These observations together show that miR-4709-3p modulate obstructive renal fibrosis by targeting the Hippo signaling pathway.

(a) Western blot analysis exhibiting protein level of LATS2, p-YAP, p-TAZ in HK-2 cells transfected with miR-4709-3p inhibitors or mimics.(b) Western blot analysis exhibiting protein level of LATS2, p-YAP, p-TAZ in HK-2 cells co-transfected with LATS2-overexpression plasmid and miR-4709-3p mimics.(Data presented as mean ± SD; *p < 0.05)

### 3.6. In-vivo inhibition of miR-4709-3p reduces the UUO-induced obstructive renal fibrosis

**Figure 6.**
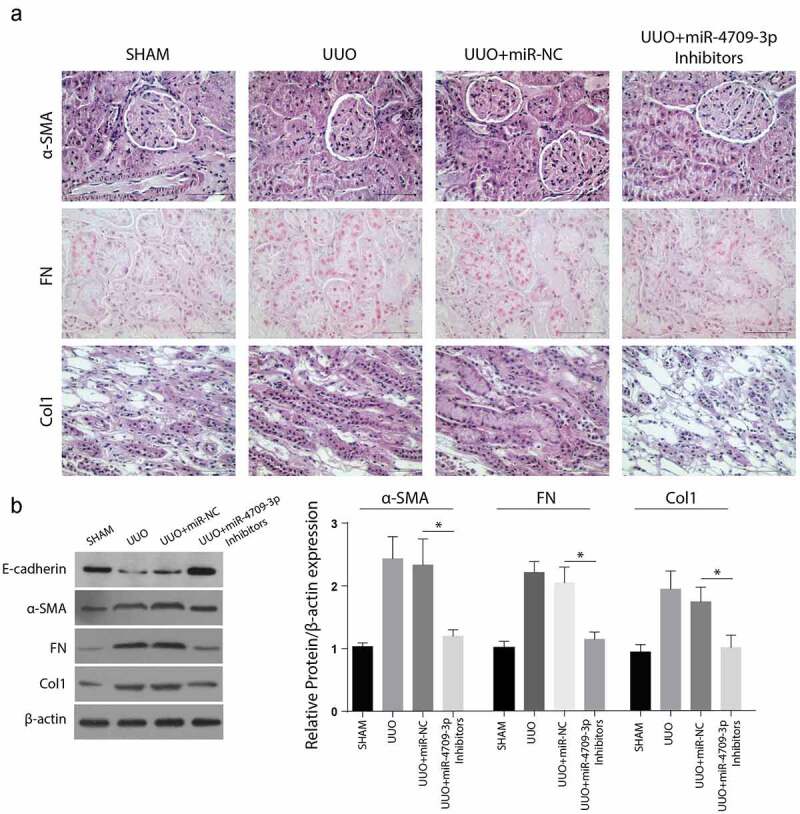
Inhibition of miR-4709-3p reduces markers of renal fibrosis in the UUO-induced in-vivo model

C57BL/6 mice were used to determine the effect of miR-4709-3p on UUO-induced obstructive renal fibrosis in vivo. According to the immunohistochemical assays results, renal fibrosis markers, Col1. Fibronectin and α-SMA were significantly reduced in the tissues from UUO+miR-4709-3p inhibitors than in the Sham, UUO, or UUO+miR-NC group shown in Figure 6(a). Similarly, the Western blot analysis exhibited significantly reduced α-SMA, FN, and Col-1, but a significantly increased E-cadherin expression levels compared to the UUO and the UUO miR-NC groups, kidney samples from UUO-induced mice model after inhibition of miR-4709-3p as shown in Figure 6(b). Taken together, these observations confirm that miR-4709-3p reduces UUO-induced obstructive renal fibrosis.

(a) IHC assay depicting protein level of renal fibrosis markers in kidney samples from UUO-induced mice model after inhibition of miR-4709-3p.(b) Western blot analysis exhibiting protein level of renal fibrosis markers in in kidney samples from UUO-induced mice model after inhibition of miR-4709-3p.(n = 6 each group; data presented as mean ± SD; *p < 0.05; scale bar = 100 µm)

## 4. Discussion

Numerous kidney diseases, e.g., chronic kidney disease (CKD), are characterized by impaired glomerular filtration. CKD is a cause great community well-being challenge associated with a significant economic burden^46^. Within the previous years, various interventional approaches have been used to leisurely CKD advancement. Such efforts strategies include intensive control of glycemia, strict blood pressure control, dyslipidemia correction, discontinuation of smoking and nephrotoxic drugs, and renin-angiotensin system blockade to minimize the pressure of glomerular capillary^47,48^. However, none of the existing measures reverses advancement to end-stage renal disease, despite the application of multifactorial therapeutic interventions, hence the need for a highly efficacious method for CKD treatment^49,50^. At the histological observation, obstructive renal fibrosis is the ultimate typical CKD result, regardless of initially reported injury^51,52^. At present, numerous reports associated the progress of miRNA in mediating obstructive renal fibrosis and Diabetic nephropathy and determined the biological functions of some miRNAs in different stages of renal fibrosis^53,54^. This study provides preclinical data to show that the use of miR-4709-3p as antifibrotic agents may weaken or stop CKD progression, even though definitive clinical trial evidence is needed.

Assessment of the profiles of miRNA expression has identified miRNAs panels, for instance, microRNA (miR)-146a, miR-215, and miR-886, together with several other miRNAs (miR-21, let-7a–g, miR-192, miR-194, miR-204, and miR-200), with enriched expression in the kidney in comparison to other important organs^55^. A study comparing miRNA expression levels in the normal versus fibrotic kidneys identified an upregulated 21 miRNAs^56^. Comparison of outcomes from animal models with ischemic injury or unilateral ureteric obstruction (UUO) with human CKD showed a common increased expression of 24 miRNAs. These proteins included: miR-214, −199, −132, −15b, −21, −25, and let-7i, indicating that these elevated miRNAs could be significant in regulating the disease process^57^. The current work also reported increased expression of miR-4709-3p, confirming that miRNAs are important in developing fibrosis in the kidney and probably attain a potential therapeutic aim for antifibrotic therapy in CKD.

MiRNAs via targeting several mRNAs and signaling pathways influence the pathogenesis of DN. This has been depicted that miR-424 in renal injury may hinder apoptosis and reduce the pathophysiological alterations in the kidney via caspase-3/ Bax/ Bcl-2^58^. Meanwhile, miR-93 considerably reduces in DN renal specimens, but miR-93 mimicking hindered TGF-β1-dependent renal fibrogenesis via binding Orai1^59^. However, it was estimated that miR-370 upregulation is positively linked with Col I, Col IV, and fibronectin increased in human RIF, DN, and CKD rats, and hindering this miR-370 improves the degradation of ECM^60^. In our study, we concluded the upregulation of miR-4709-3p to be associated with RIF and CKD.

TGF-β is also essential in renal fibrosis induction. According to studies, several miRNAs which have roles in renal fibrosis modulation are controlled by TGF-β^61^. TGF-β1 increases miR-21, miR-150, miR-143, miR-192, and miR-377 but suppresses miR-200 and miR-29 in animal models or patients with renal fibrosis^62^. In this study, the findings showed increased miR-4709-3p in the kidney during UUO-induced renal fibrosis and TGF-β induced renal injury in HK-2 cells. The findings also agree with a previous report that noted that TGFβR2 expression level directly correlates with TGF-β response and essentially induces renal fibrosis after kidney injury^63^.

The Hippo pathway affects various biological processes in the growth and development of tissues and organs, and LATS1/2 forms one of its main components^22^. LATS 1/2 precisely targets the YAP/TAZ, which are thoroughly involved in CKD progression^64^. Studies have reported significantly increased TAZ and YAP protein levels in the kidneys of UUO mice and linked with ECM accumulation and stiffness of the kidney^65^. In agreement with these previous studies, our findings also confirmed a significantly increased p-TAZ, p-YAP, and LATS2 in renal fibrosis mice, following inhibition of miR-4709-3p.

## 5. Conclusion

In conclusion, this study’s results demonstrated that aberrant miR-4709-3p expression plays an essential function in renal fibrosis progression. Briefly, it was determined that miR-4709-3p is increased in the human DN and RIF specimens, TGF-β1 induced HK-2 cells, and the UUO-induced obstructive renal fibrosis model. Furthermore, silencing miR-4709-3p reduced renal fibrosis markers, while overexpression of miR-4709-3p exerted opposite effects. Meanwhile, it was verified that miR-4709-3p targets 3ʹUTR of LAST2, thus enhance the expression of renal fibrosis markers and accelerates obstructive renal fibrosis advancement via the Hippo signaling pathway. Therefore, miR-4709-3p inhibition may be used for renal fibrosis therapy (as shown in the graphical abstract).
